# Whole Body Vibration Training Has No Effect on Vascular Endothelial and Inflammatory Markers in Young Healthy Women

**DOI:** 10.3390/jcm13144228

**Published:** 2024-07-19

**Authors:** Halina Gattner, Justyna Adamiak, Olga Czerwińska-Ledwig, Sylwia Mętel, Magdalena Kępińska-Szyszkowska, Anna Piotrowska

**Affiliations:** 1 Faculty of Physiotherapy, University of Physical Education in Krakow, Jana Pawła II Avenue 78, 31-571 Krakow, Poland; 2 Institute of Applied Sciences, Faculty of Physiotherapy, University of Physical Education in Krakow, Jana Pawła II Avenue 78, 31-571 Krakow, Poland; 3 Department of Chemistry and Biochemistry, Faculty of Physiotherapy, University of Physical Education in Krakow, Jana Pawła II Avenue 78, 31-571 Krakow, Poland

**Keywords:** whole body vibration training, WBVT, inflammation, angiogenesis, eNOS

## Abstract

**Background:** The aim of the study was to comparatively assess the impact of single and repeated whole body vibration training (WBVT) and training without vibration on changes in the concentration of vascular endothelial growth factor (VEGF), endothelial nitric oxide synthase (eNOS), and high-sensitivity C-reactive protein (hsCRP) in healthy, young, non-training women. **Methods:** The study involved 46 women (age 20.48 ± 1.72 years), who were divided into three groups: the experimental group participating in WBVT (EVG, *n* = 17); the comparison group performing the same exercises but without the vibration factor (EXG, *n* = 12); and the control group, which did not participate in any training (CON, *n* = 17). The program included participation in 36 training sessions conducted over 12 weeks, with a frequency of 3 times per week. In the EVG and EXG groups, venous blood was collected before and after the first and last training sessions, while in the CON group, blood was collected twice at a 3-month interval. **Results:** No significant changes were observed in the concentrations of the studied markers either after a single or repeated training session in both experimental groups (*p* > 0.05). **Conclusions:** The proposed WBVT protocol appears to be a safe form of exercise that does not induce negative inflammatory reactions. The applied vibration stimulus combined with physical exercises did not initiate pro-angiogenic processes or stimulate eNOS activity in healthy women, suggesting that similar studies should be conducted in individuals with circulatory problems or chronic inflammatory diseases.

## 1. Introduction

Exercise-induced angiogenesis is a key adaptive process involving the formation of new capillaries from existing vessels to enhance the delivery of oxygen and nutrients to active skeletal muscles. During exercise, increased blood flow and shear stress lead to the elevation of several vasodilatory substances, including nitric oxide (NO), which regulates the expression of vascular endothelial growth factor (VEGF), the primary pro-angiogenic factor present in muscle fibers and endothelial cells. NO can be produced by endothelial nitric oxide synthase (eNOS), located in the endothelium, among other places, or by neuronal nitric oxide synthase (nNOS), primarily found in muscle fibers [1,2]. Oxidative stress can both trigger and exacerbate inflammatory processes in tissues. Factors that may contribute to oxidative stress resulting in cellular component damage include excessive physical exertion and aging. Increased release of reactive oxygen and nitrogen species has been observed in conditions associated with chronic inflammation, including cardiovascular diseases [3]. One of the significant markers of the inflammatory response is C-reactive protein (CRP), a glycoprotein produced by liver cells in response to inflammatory cytokines, belonging to the group of acute-phase proteins. High sensitivity CRP (hsCRP) testing is used to predict cardiovascular events [4]. Some possible explanations for the beneficial effects of physical exercise in reducing the risk of cardiovascular diseases include the reduction of systemic inflammation and oxidative stress, regulation of blood lipid and glucose levels, increased insulin sensitivity, and structural and functional remodeling of the cardiovascular system, including increased capillary density in muscles [1,5,6].

Epidemiological data suggest that physical activity levels are inversely correlated with markers of inflammation, including CRP [7,8,9]. However, studies on physical training have not consistently shown that exercise reduces CRP levels in women. For instance, Maierean et al. found no significant reduction in CRP levels among women following exercise interventions [10]. Similarly, Campbell et al. [11] observed no changes in CRP levels in both men and women leading a sedentary lifestyle after a 12-month aerobic training program, despite significant improvements in aerobic capacity and reductions in body weight and fat mass. This suggests that exercise may play a greater role in individuals with higher initial levels of inflammation, such as obese individuals. These findings are consistent with results from other studies focusing solely on exercise [12,13]. Some studies suggest that the reduction in CRP levels following exercise intervention may be due to the associated weight loss [10,12]. On the other hand, findings from Daray et al. [14] suggest that a combination of endurance and resistance training may be an effective method for lowering plasma CRP levels in young, healthy women, regardless of changes in aerobic capacity or body composition. Cunha et al. [15] and other studies by Tomeleri et al. [16] observed a reduction in CRP levels after a 12-week resistance training program in older women. Similar results of CRP reduction following strength and aerobic training in a mixed group were obtained by Martins et al. [17]. According to the authors, the decrease in hs-CRP levels is associated with increased strength and fat loss.

Researchers report that improvement in endothelial function can occur as a result of strength, endurance, and mixed training [18,19,20]. Systematic training enhances the bioavailability of NO by modulating various mechanisms, including increasing eNOS expression, boosting the efficiency of the antioxidant system, and reducing oxidative stress and inflammatory markers [21,22,23]. Ribeiro et al. [24] studied the impact of a single bout of resistance exercise at varying intensities on the mobilization of circulating endothelial progenitor cells and angiogenic factors within 24 h in women. The results indicate an increase in these markers, including VEGF, with the highest exercise intensities promoting the greatest increase in circulating indicators. In other studies conducted on women aged 30–40 years, an increase in VEGF levels was also noted after 8 weeks of aerobic exercise [25]. However, after 12 weeks of Nordic walking training, no changes were observed in eNOS activity in healthy postmenopausal women [26]. Ratajczak et al. [27] conducted an interesting study comparing the effects of a 3-month stationary bike training program on women aged 40–60 years with obesity and women with normal body weight. Following the intervention, obese women experienced, among other things, weight loss, improved lipid metabolism, and a decrease in the inflammatory marker CRP. No changes in eNOS activity were observed in either group. Other studies have shown that in obese women, both endurance and endurance-strength training improve lipid metabolism, but only aerobic exercises increased eNOS activity [28]. However, the mechanism related to eNOS activation induced by training remains unclear. It is known that shear forces increase during physical exercise and are recognized as the main stimulus for eNOS activation [21]. 

In this study, we specifically chose to examine women for several reasons. Firstly, women often exhibit different physiological responses to exercise compared to men, including differences in hormone levels, body composition, and cardiovascular function. These differences can significantly impact the outcomes of exercise interventions, making it essential to study women separately to understand these unique effects [29,30]. Furthermore, cardiovascular diseases, although often associated with men, are a leading cause of mortality among women [31]. Women also tend to experience a higher prevalence of obesity and related metabolic conditions, which are risk factors for cardiovascular diseases [32].

Whole-body vibration training (WBVT) utilizes a controlled vibratory stimulus during static or dynamic physical exercises performed on a specialized platform [33]. Mechanical vibrations stimulate muscle spindles, which respond with tonic vibration reflexes (TVR), thereby increasing muscle activity [34,35]. This training has characteristics of resistance training, as the applied vibrations lead to increased gravitational load and reflexive muscle contractions [36]. The maximum single exposure to whole-body vibrations on the platform typically lasts 30 min [37]. WBVT can serve as an alternative to traditional exercises, allowing for effective training with minimal physical effort, particularly for individuals who have difficulty moving (e.g., those with cognitive impairments, musculoskeletal disorders, post-injury and post-trauma, obesity) or those with cardiovascular diseases and chronic conditions who cannot tolerate higher levels of activity. Many researchers highlight the stimulating impact of WBVT on the musculoskeletal system, resulting in increased muscle strength and power [38,39,40,41] and bone mineral density [42,43,44]. Beneficial changes are also observed in the circulatory system, such as increased peripheral blood flow and better muscle oxygenation [45,46,47]. Available scientific studies confirm that vibration training regulates immune functions and exerts an anti-inflammatory effect [48,49,50]. However, the impact of WBVT on angiogenesis markers such as VEGF and eNOS, and the inflammation marker CRP in young and healthy women has not been thoroughly described in the literature.

Therefore, the aim of this study was to assess the impact of single and 12-week WBVT and standard training without vibration stimulus on the levels of endothelial function markers and CRP protein concentration. We hypothesized that WBVT would have a more pronounced positive effect on CRP, VEGF, and eNOS levels than standard training alone. Given that cardiovascular diseases are increasingly prevalent among younger individuals [51], this study provides significant information regarding the use of WBVT in the prevention and treatment of these conditions in a younger population of women. WBVT appears to be an attractive form of exercise, especially for individuals who prefer short-duration efforts and those with lower levels of physical activity.

## 2. Materials and Methods

### 2.1. Participants

The study involved healthy, non-training women (*n* = 54, mean age 20.48 ± 1.72 years) who met the following inclusion criteria: age between 18–25 years; female gender; written consent to participate in the study; low (insufficient) level of physical activity as determined by the International Physical Activity Questionnaire—IPAQ [52]; body mass index (BMI) in the range of 18.5–24.9 [53]; diet consistent with the recommendations of the Polish Institute of Food and Nutrition [54]; no contraindications to WBVT confirmed by medical qualification [55]. Exclusion criteria included: smoking; use of hormonal contraception; diagnosed polycystic ovary syndrome or anovulatory cycles; special and elimination diets within 3 months prior to the study; regular intake of antioxidant vitamins and other dietary supplements at least one month before the start of the study. The characteristics of the selected groups are presented in Table 1.

**Figure 1:**
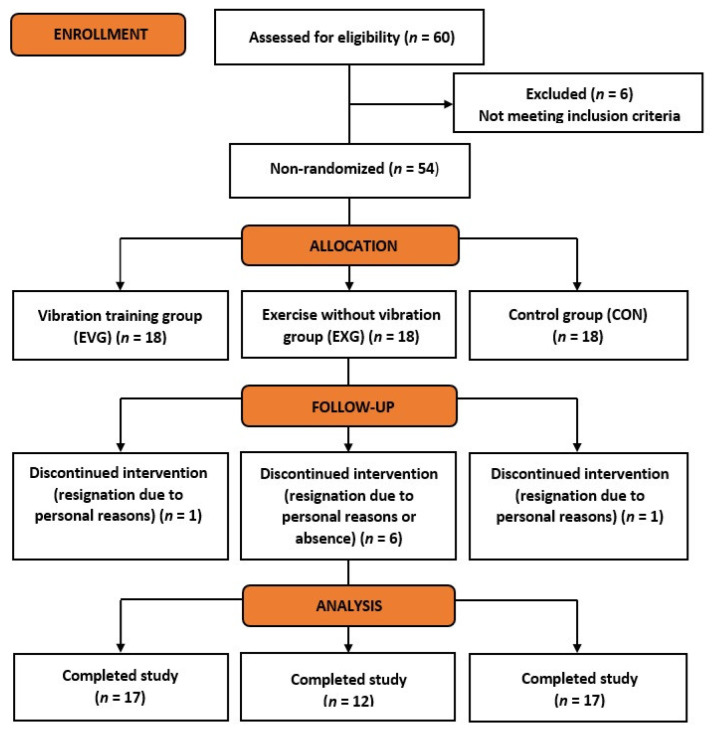
Study flow diagram.

From the 54 women enrolled and qualified for the study, three groups of 18 women each were distinguished: the experimental group with physical training on a vibration platform (EVG), the comparison group performing the same exercise program but without the vibration factor (EXG), and the control group, which did not undergo any physical activity (CON). Allocation of subjects into different groups was done through volunteer sampling—the participants were recruited via posted advertisement at the university and assigned to the group to which they volunteered. The study was completed by 17 women in EVG and CON, and 12 in EXG. The scheme of the recruitment process is shown in Figure 1.

The study was conducted in accordance with the Declaration of Helsinki. Approval for this project was obtained from the Bioethics Committee at the District Medical Chamber in Krakow (approval number 224/KBL/OIL/2016). The project was registered in the clinical trial database (trial ID: ACTRN 12621000114842). The project was funded by grants No. 101/MN/KK/2017 and 101/MN/INB/2017 awarded by the University of Physical Education in Krakow. Participation in the research project was voluntary. The women were informed about the purpose of the study, the methodology used, potential side effects, and the possibility of withdrawing from the study at any time without providing a reason. 

### 2.2. Study Protocol

The study protocol was divided into two phases: an initial and a main phase. At the start, participants in each group underwent interviews, medical examinations, body composition assessments, nutritional analyses, and evaluations of physical activity levels. The main phase involved a 12-week training program for both the experimental and comparison groups, with blood samples collected at four time points: before the first training session, after the first training session, before the final training session, and after the final training session. The preliminary phase was conducted within one month prior to the main phase. In the control group, blood samples were collected twice, three months apart. The research was executed over two stages across two consecutive years. In the first stage, the experimental group exercised on a vibration platform while the control group did not undergo any intervention. In the second stage, the comparison group followed the same exercise regimen without the vibration component. Recruitment and selection of participants for each group were conducted separately from the pool of registered volunteers.

### 2.3. Anthropometric Measurements

Before the study began, the following measurements were taken for the women: body mass (BM), percentage of body fat (PF), fat mass (FM), fat-free mass (FFM) and body mass index (BMI) using the bioelectrical impedance method with a TANITA BC 418 MA body composition analyzer (Tanita, Tokyo, Japan). Body height (BH) was measured using an anthropometer.

### 2.4. Nutritional Analysis and Physical Activity Level

Before the experiment began, the participants’ dietary habits were assessed using a food diary. The women recorded the type and amount of food and beverages consumed over four weekdays and one weekend day (free from classes or work). The Dieta 5 computer program (Institute of Food and Nutrition, Warsaw, Poland) [54] was used for the nutritional analysis. The average energy value of the consumed food and the proportion of basic nutrients (carbohydrates, proteins, fats) were calculated. To assess the level of physical activity, the short version of the International Physical Activity Questionnaire (IPAQ), which includes 7 questions about physical activity over the past week, was used [52].

### 2.5. Venous Blood Collection and Biochemical Assays

Blood was drawn from the participants while fasting in the morning hours in a seated position by a qualified laboratory diagnostician according to the current standards. A BD Vacutainer vacuum system (Becton Dickinson, Franklin Lakes, NJ, USA) was used for blood collection, and blood was collected into tubes with a clot activator. The blood draws were scheduled to occur during the follicular phase of the menstrual cycle. In the control group, blood was collected twice at a 3-month interval. In the experimental and comparison groups, venous blood was drawn four times: immediately before and after the first training session and immediately before and after the last training session.

After collection, the blood was allowed to clot in the tube, then the sample was centrifuged according to accepted standards. The serum was pipetted into Eppendorf tubes and stored at low temperature (−80 °C, ULF 390 Arctiko Dairei, Esbjerg, Denmark) until analysis. Serum concentrations of vascular endothelial growth factor (VEGF) [pg/mL] and endothelial nitric oxide synthase (eNOS) activity [U/mL] were measured using enzyme-linked immunosorbent assay (ELISA) with high-sensitivity reagent kits from SunRed Bio (Wuhan, China). An ELISA microplate reader (ChroMate 4300, Awareness Technology Inc, Palm City, FL, USA) was used to read the results at a wavelength of 450 nm. The sensitivity of the tests was 18.827 pg/mL for VEGF and 0.45 U/mL for eNOS. The concentrations of hs-CRP protein were determined in an external medical diagnostic laboratory using immunoturbidimetry on a Roche Cobas 6000 device (Basel, Switzerland).

### 2.6. Exercise Program

**Figure 2:**
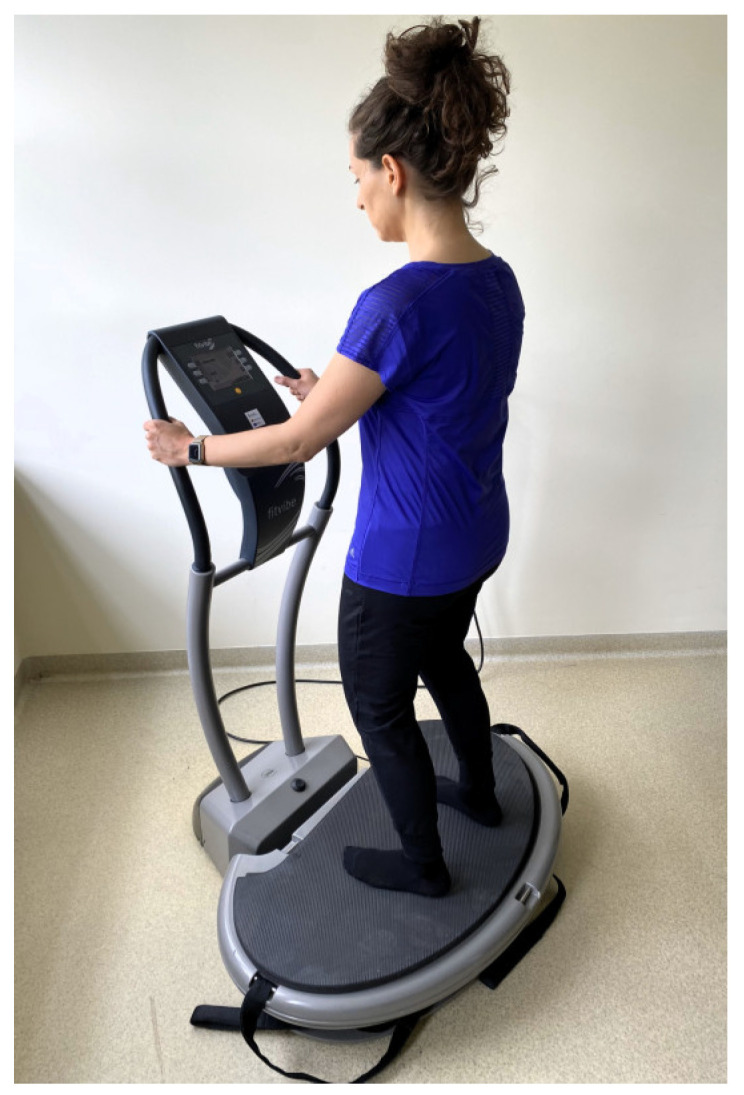
Standing position on the vibration platform.

**Figure 3:**
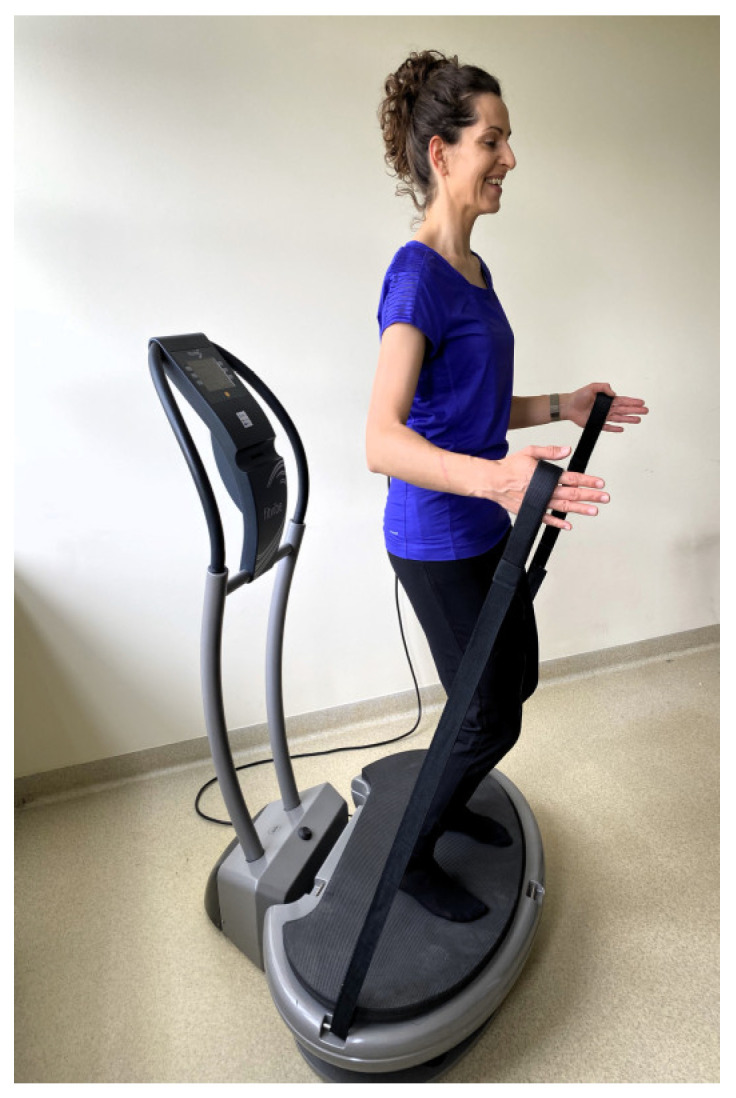
Standing position on the vibration platform with holding taut bands.

**Figure 4:**
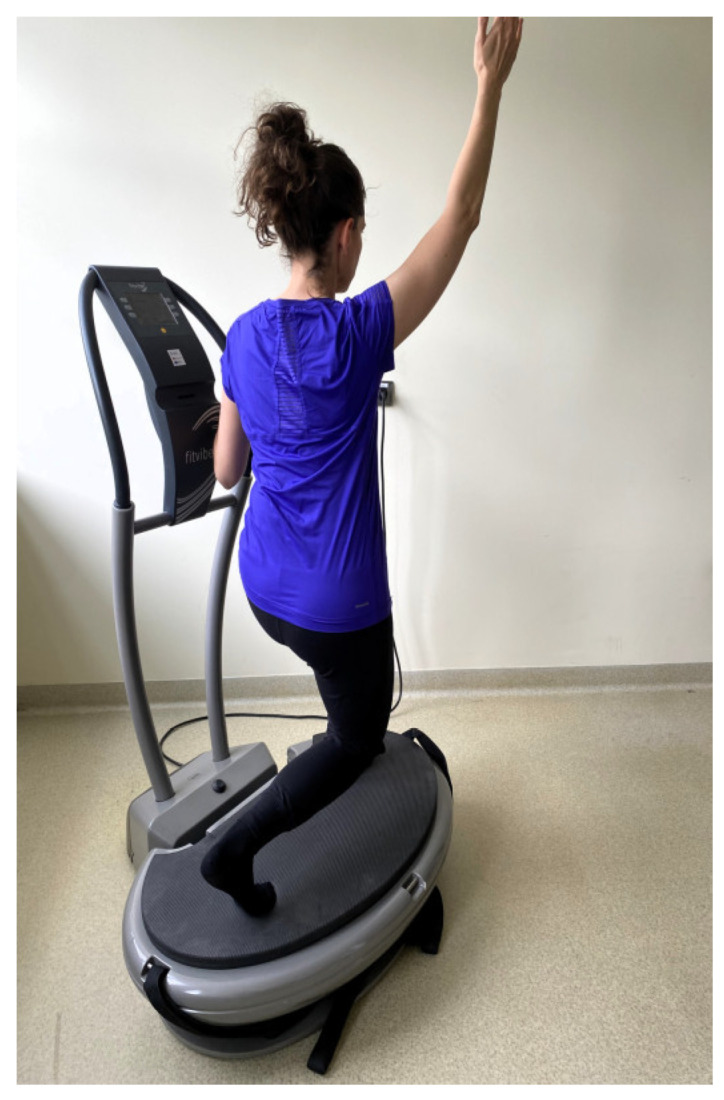
Dynamic side squats on the vibration platform.

The women in the EVG and EXG groups participated in 36 training sessions over 12 weeks, with a frequency of 3 times per week. WBVT was conducted as individual sessions with an instructor using a Fitvibe Excel Pro linear vibration platform (Gymna Uniphy, Bilzen, Belgium). To minimize the transmission of vibrations towards the head during the exercises, body weight was shifted onto the forefoot. The training protocol included a warm-up consisting of 3 exercises performed in a standing position, with 6 repetitions each. In the main part of the session, women in the EVG performed 6 exercises in a standing position on the vibration platform:

- maintaining a standing position on the platform (Figure 2);
- dynamic half-squats;
- maintaining a standing position on the platform with the back to the platform while holding taut bands attached to the base (Figure 3);
- dynamic side squats on the platform in a lunge position with the right lower limb while simultaneously raising the opposite upper limb;
- dynamic side squats on the platform in a lunge position with the left lower limb while simultaneously raising the opposite upper limb (Figure 4);
- maintaining a half-squat position.

Each exercise on the platform was performed for 1 min. There was a 1-min break between each exercise, during which the women walked around the room. Dynamic squats were performed to the rhythm of a metronome at a pace of 25 movements per minute. The vibration amplitude (2 mm) and the duration of exercises and breaks between them (1 min) remained constant throughout the training period. During the first 12 training sessions, the vibration frequency was 40 Hz, then it increased by 5 Hz after every subsequent set of 12 sessions (40–45–50 Hz). 

The final part included 3 exercises performed on a mat, with 6 repetitions each. The EXG followed the same training scheme without using the vibration platform in the main part, conducted as group sessions with 3–4 participants (except for the first and last sessions, which were conducted individually). The detailed methodology of the exercises is described in our previous article [56].

### 2.7. Statistical Analysis

The sample size was determined a priori (G*Power version 3.1.9.7; Dusseldorf, Germany) The following assumptions were taken into account: test family = F tests; type of power analysis = computed required sample size—given α, power and effect size; effect size f: 0.25; α error probability: 0.05; power: 0.85; number of groups: 3. The required total sample size was 36 participants (12 per group).

Statistical analysis was conducted using SPSS Statistics 24 (IBM). Results are given as mean values ± standard deviation (SD), minimum and maximum value. For tests results, 95% confidence interval (CI) was given as an additional measure of dispersion. The normality of variable distributions was checked using the Shapiro-Wilk test. Based on the distribution type, differences in physical characteristics and nutrition between groups at the baseline of the study were examined using one-way analysis of variance (ANOVA) or the Kruskal-Wallis test, with corresponding post-hoc tests (Sidak or Dunn’s test). To verify differences between the analyzed biochemical indices and the time of their measurement (measurement points 1–4), differences between groups in a given measurement, and interaction effects of measurement time and group membership, a two-way mixed model ANOVA (univariate mixed model ANOVA) was conducted. To verify the significance of the observed effects, multiple comparisons were performed using Sidak’s and Fisher’s Least Significant Difference (LSD) tests. VEGF scores due to significant baseline discrepancies were tested with the ANCOVA where the baseline VEGF score was considered covariante. The significance level was set at α = 0.05. The effect size was assessed using the Eta squared coefficient (η^2^) and interpreted as follows: 0.1—small; 0.25—medium; 0.37–large size effect [57].

## 3. Results

### 3.1. Characteristics of the Participants

In the baseline study, there were statistically significant differences between the groups regarding body mass and fat-free mass. Women in EVG had lower body mass (BM) and fat-free mass (FFM) compared to those in CON [Table 1].

### 3.2. Vascular Endothelial Growth Factor (VEGF)

The results of the VEGF concentration test are shown in Table 2. Regarding VEGF, a significant main effect of the group was observed (*p* = 0.003). Multiple comparisons indicated that individuals in EVG had higher VEGF levels than those in EXG in measurements taken both before and after the first and last vibration training sessions (*p* < 0.001) [Table 3].

Assessing the long-term effects, a significant main effect of the group was also found (*p* = 0.003). Post-hoc test results showed that EVG significantly differed from EXG in both the first and third measurements, with higher VEGF values (*p* = 0.002) [Table 4].

Due to significant differences in baseline VEGF concentrations, as indicated by the conducted analysis (two-way ANOVA, Table 3 and Table 4), another statistical test was performed. ANCOVA was used, with baseline VEGF concentrations indicated as covariates.

The ANCOVA results indicated that there were existing differences between the results obtained in the study (*p* < 0.001; η^2^ = 0.812). The effect size of this difference should be interpreted as large. However, after adjusting and indicating the baseline measurement as a covariate, no differences were indicated between the groups (*p* < 0.446; η^2^ = 0.004), meaning the change in VEGF concentration was identical after a single exercise session regardless of whether it was performed on a vibration platform or not. An analogous test was conducted comparing the effect of the last session. Without considering the covariate, significant differences were indicated (*p* < 0.001; η^2^ = 0.865), whereas after the adjustment, no significant differences were indicated (*p* = 0.989; η^2^ < 0.01). 

In the next step, the impact of a series of proposed exercises performed on the platform (EVG) and without such a platform (EXG) on VEGF concentration was checked, and the results obtained for the active groups were compared with the control group (CON). As indicated in Table 3, the results analyzed with ANOVA showed significant differences, but after the correction (ANCOVA test), these findings were not confirmed. Without the adjustment: I vs. III showed significant differences with a large effect size (*p* < 0.001, η^2^ = 0.830), whereas after the adjustment, the difference between the groups was not significant (*p* = 0.818; η^2^ = 0.002). 

### 3.3. Endothelial Nitric Oxide Synthase (eNOS)

The results of the eNOS concentration test are shown in Table 5. For eNOS, no significant main effects were found for measurement time, group, or the interaction between measurement time and group (*p* > 0.05) [Table 3 and Table 4].

### 3.4. High-Sensitivity C-Reactive Protein (hsCRP)

The results of the hsCRP concentration test are shown in Table 6. The analysis of hsCRP did not reveal any significant main effects for measurement time, group, or the interaction between measurement time and group (*p* > 0.05) [Table 3 and Table 4].

## 4. Discussion

In the present study, no changes in eNOS activity were observed in the EVG and EXG groups in response to either single or repeated training sessions. There is a lack of scientific reports in the literature evaluating eNOS activity in response to WBVT. To our knowledge, this is the first study to analyze the activity of this enzyme in response to vibration exercises. Available reports indicate an increase in nitric oxide (NO) concentration following WBV application [58,59], as well as local vibration application [60], which may also suggest increased expression of the endothelial isoform of NOS. Furthermore, the results of this study do not confirm the impact of either single or repeated WBVT on hsCRP levels, which supports the safety of this form of exercise and its potential use in patients with chronic inflammatory diseases. The applied WBVT protocol was also insufficient to initiate angiogenic processes in healthy, young women, as evidenced by the lack of changes in VEGF concentration.

The results of our own study did not demonstrate an increase in eNOS. The impact of WBVT on vascular function is not fully understood; however, it is believed that WBVT, similar to other physical exercises, increases shear stress on vascular endothelium, leading to increased activity of eNOS and enhanced production of NO, resulting in improved blood flow [58,61,62]. Vascular wall stress is also influenced by so-called tensile stress generated by arterial pressure. These two biomechanical forces significantly affect eNOS expression in vessels [63,64]. Vibrations transmitted to the human body cause alternating contractions and relaxations of muscles, which act like a pump on the circulatory system. This results in the dilation of blood vessels, increased blood perfusion through tissues, and better oxygen and nutrient supply to muscles [62,65]. Moreover, WBV, through mechanical stimulation, affects vascular functions, leading to reduced arterial tension and stiffness [66,67,68].

Referring to the results of a meta-analysis conducted in 2015 [69], the lack of impact of WBV on eNOS activity observed in this study may be due to the platform parameters used. In the group of women subjected to WBVT, a linear platform with settings in the range of 40–50 Hz was utilized. According to Games et al. [69], blood flow increases primarily with the application of alternating vertical vibrations at lower frequencies in the range of 5–25 Hz generated by axial platforms. The authors of the meta-analysis attribute this to the different rates of muscle contraction. The use of WBV with lower frequency values may result in longer breaks between successive muscle contractions, thereby leading to greater blood perfusion. From the perspective of understanding these issues, it would also be crucial to complement the conducted studies with measurements of nitric oxide concentration. Additionally, as reported by Green et al. [21], achieving improvements in endothelial function in healthy and asymptomatic individuals may require higher training loads compared to individuals with endothelial dysfunction.

The present study showed no changes in the VEGF level in both the vibration training group and the standard training group. As other studies have idenitfied the primary regulators of VEGF secretion in response to physical exercise are shear stress generated during muscle activity and oxygen availability [2,70]. VEGF is classified as a factor that stimulates angiogenesis, influencing the increase in the number and permeability of blood vessels. Additionally, it participates in the processes of vasodilation and prevents platelet aggregation. All these actions enable increased blood flow and efficient oxygen transport to tissues [71]. Both aerobic and resistance exercise promote the growth of pro-angiogenic factors and the activation of their receptors in the body [72,73,74,75]. WBVT can be considered a form of resistance exercise, involving the adaptation of the exerciser’s body to the repeated oscillations of the platform. Vibrations with appropriate parameters increase the effect of gravitational forces due to the large accelerations cyclically transmitted to the body. This phenomenon can be compared to traditional strength training, where gravitational overload is achieved by using additional weights, such as lifting weights [36,76].The study by Jawed et al. [49] conducted with healthy men demonstrated that an 8-min exposure to WBV in a standing position on a platform (35 Hz, 4 mm) leads to an increase in VEGF and TNF-α, which synergistically act to promote angiogenesis. In our study, no changes in VEGF levels were observed either after the first or the last training session. One of the reasons for the discrepancy between the results of the analyzed indicators could be the biological material used for measurement (serum vs. plasma). 

Available scientific reports confirm that tissue hypoxia is a stimulating factor for the angiogenesis process [77,78]. A review of the literature reveals studies analyzing the impact of hypoxia combined with physical exercise on VEGF [79,80]. Chih-Min et al. [79] evaluated the effects of single WBVT and WBVT combined with local hypoxia using blood flow restriction (BFR) cuffs on the thigh muscles on VEGF levels in healthy, physically inactive men. Participants assumed a static squat position on the vibration platform at a 100-degree knee flexion angle for 10 sets of 1 min each, with 1–2-min breaks between sets (frequency 26 Hz, amplitude 4 mm). WBVT alone did not cause significant changes in VEGF concentration, but a significant increase was observed after WBVT combined with BFR. In other studies, involving healthy men, the effects of whole-body vibration and normobaric hypoxia during intense exercise on a cycle ergometer on changes in angiogenic markers were analyzed. The results showed VEGF expression following physical exercise combined with exposure to mechanical vibrations at an amplitude of 4 mm and a frequency of 30 Hz [80]. Comparing the results obtained by the aforementioned authors with our own, it should be emphasized that these studies differed significantly in terms of the type of exercises performed and the intensity of the training. Factors that may have influenced the results also include the duration of a single training session and the duration of a single exposure to vibrations, which were likely insufficient to initiate pro-angiogenic processes.

The analysis of our own results also did not show changes in VEGF concentration under the influence of 12-week WBVT. It is suspected that this was due to the high inter-individual variability in baseline VEGF levels in the study groups. It should also be noted that the VEGF concentration measured in blood serum was within the reference range for all women participating in the study [81]. In the study conducted by Beijer et al. [82] involving young, healthy men, it was found that adding whole-body vibration to resistance training did not stimulate signaling pathways promoting angiogenic processes in skeletal muscles. The frequency of the axial platform vibrations used in the study increased by 5 Hz weekly, from 20 to 40 Hz, while the amplitude remained constant at 6 mm. The authors explain that adding vibration to strength training leads to reduced endothelial cell proliferation, likely due to decreased release or expression of VEGF.

In our own research, as well as available scientific literature indicates that repeated WBVT does not negatively affect inflammation markers [83,84]. On the other hand, others researchers have demonstrated immunomodulatory and anti-inflammatory effects [50,85]. According to a systematic review from 2022, WBV can be useful as a non-pharmacological method for treating inflammatory conditions in both healthy populations and individuals with chronic diseases. This method may be particularly beneficial for patients for whom the use of oral anti-inflammatory drugs is contraindicated due to adverse effects [86]. The possible mechanism by which WBVT affects inflammatory responses can be attributed to its ability to stimulate sensory elements that promote systemic reactions [87]. However, there is no consensus in the literature regarding the duration, frequency, and amplitude of vibrations required to achieve the desired biological effects. Rodriguez-Miguelez et al. [85] demonstrated a decrease in CRP and TNFα levels in healthy older adults after 8 weeks of WBVT conducted twice a week at frequencies of 20–35 Hz and an amplitude of 4 mm. Similarly, Mohammed and Mawad [50] found that 12 weeks of WBVT performed three times a week with parameters of 27–35 Hz and 2 mm amplitude significantly reduced CRP, TNFα, and IL-6 levels compared to aerobic training in older men. In contrast, Watanabe et al. [84] found that incorporating whole-body vibrations with increasing frequency and low amplitude (30–35–40 Hz) into a 4-week aerobic exercise protocol did not produce an anti-inflammatory effect in healthy young individuals. Additionally, Theodorou et al. [83] reported no changes in CRP levels in middle-aged healthy women following either a single exposure to WBV or a 2-month vibration training program conducted three times a week on an axial platform (frequency 20–25 Hz, amplitude 6 mm).

### Limitations and Strengths of the Study

The main limitations of the conducted study include the relatively low number of participants in the comparison group compared to the experimental and control groups, and the lack of random assignment to the groups (participants were healthy volunteers from the student community rather than a random sample of the general population of young women). Furthermore, the study did not include a group subjected solely to whole-body vibrations. Such an intervention could provide significant information regarding the discussed topics. Another limitation was that measurements were conducted at only one time point post-exercise. The levels of the studied markers might have varied if blood samples had been additionally collected at 15 and 30 min post-training. The study participants were young, healthy women. Conducting similar studies in older individuals or those with chronic conditions, such as cardiovascular diseases, might yield more pronounced changes in the biochemical markers analyzed.

A strong point of the study is that all participants were in the same phase of their menstrual cycle during the measurements. There are often concerns about including women in studies due to the various physiological changes that occur throughout the menstrual cycle. Additionally, this study has several other strengths. The 12-week intervention duration provides a comprehensive understanding of the long-term effects of WBVT on biochemical markers. Multiple measurements (before and after the first and last training sessions) allow for observing dynamic changes and precisely determining training effects.

Including experimental and control groups facilitates comparisons between WBVT, other forms of training, and no intervention. Analyzing various markers, such as eNOS, hsCRP, and VEGF, offers a holistic assessment of the impact on vascular health and inflammation. Precise control of training protocols and standardized study conditions (consistent nutrition and physical activity guidelines) enhance result reliability and reproducibility. Focusing on young, healthy women helps assess WBVT effects without the confounding impact of other illnesses, simplifying the interpretation of findings. These strengths significantly contribute to the study’s credibility and accuracy, providing valuable insights into the effects of whole-body vibration training on health.

## 5. Conclusions

Physical exercises performed in combination with whole-body vibration (WBVT) and without the vibration factor did not affect the concentration of endothelial function markers or the level of high-sensitivity C-reactive protein (hsCRP). The proposed WBVT protocol appears to be a safe form of exercise that does not induce negative inflammatory responses. The applied vibration stimulus, combined with physical exercises, did not initiate pro-angiogenic processes or stimulate eNOS activity in healthy, young women. Future studies involving individuals with cardiovascular diseases and chronic inflammatory conditions could provide valuable insights regarding the analyzed markers.

Although our initial hypotheses were not confirmed, the conclusions from this study suggest the potential for using higher exercise intensity or increased platform parameters to determine the positive impact of WBVT on angiogenesis and inflammatory markers in a young population. Considering the well-known cardiovascular benefits of regular physical activity, there is a need for a deeper understanding of WBVT’s potential. This could guide cardiovascular disease prevention strategies and the treatment of various clinical conditions in individuals with low activity levels.

## Figures and Tables

**Table 1:** Characteristics of the subjects.

		M	SD	95% CI	95% CI	Min	Max	*p* Values (η^2^)
Age [years]	EVG	21.65	1.80	20.72	22.57	19.00	25.00	0.064 (0.097)
	EXG	20.17	1.75	19.06	21.28	19.00	25.00	
	CON	19.53	0.72	19.16	19.90	19.00	21.00	
	total	20.48	1.72	19.97	20.99	19.00	25.00	
BH [cm]	EVG	162.76	7.51	158.90	166.63	151.00	173.00	0.187 (0.095)
	EXG	164.67	5.94	160.89	168.44	157.00	173.00	
	CON	167.24	4.56	164.89	169.58	160.00	173.00	
	total	164.91	6.32	163.04	166.79	151.00	173.00	
BM [kg]	EVG	56.57	7.18	52.88	60.26	41.90	70.40	0.029 (0.137)
	EXG	59.43	6.04	55.59	63.26	50.20	69.10	
	CON	63.29 *	8.71	58.81	67.77	51.20	77.90	
	total	59.80	7.92	57.44	62.15	41.90	77.90	
BMI [kg/cm^2^]	EVG	21.31	1.87	20.35	22.28	18.40	25.20	0.351 (0.052)
	EXG	22.02	2.91	20.17	23.87	18.20	26.80	
	CON	22.57	2.44	21.32	23.83	19.50	28.60	
	total	21.96	2.40	21.25	22.67	18.20	28.60	
PBF [%]	EVG	23.04	6.11	19.89	26.18	9.90	31.00	0.351 (0.068)
	EXG	25.62	4.14	22.99	28.25	18.50	31.50	
	CON	26.25	5.76	23.29	29.22	18.10	35.90	
	total	24.90	5.61	23.23	26.56	9.90	35.90	
FM [kg]	EVG	13.34	4.54	11.01	15.67	4.20	20.00	0.119 (0.100)
	EXG	15.40	3.85	12.96	17.84	9.30	21.80	
	CON	17.03	5.89	14.00	20.06	9.30	27.90	
	total	15.24	5.08	13.73	16.75	4.20	27.90	
FFM [kg]	EVG	43.24	3.81	41.28	45.19	37.80	52.20	0.013 (0.143)
	EXG	44.03	2.84	42.22	45.83	40.20	48.50	
	CON	46.25 *	3.24	44.58	47.91	40.30	51.30	
	total	44.55	3.56	43.50	45.61	37.80	52.20	

EVG—vibration training group; EXG—exercise without vibration group; CON—control group; body height (BH); body mass (BM); body mass index (BMI); percentage body fat (PBF); fat mass (FM); fat free mass (FFM); M—mean value; SD—standard deviation; 95% CI—upper and lower bound of the confidence interval; Min—minimum value; Max—maximum value; η^2^ = eta squared; * Significant difference (*p* < 0.05) between the EVG/CON.

**Table 2:** Basic descriptive statistics of the VEGF variable.

		M	SD	95% CI	95% CI	Min	Max
VEGF I	EVG	240.48	94.40	190.18	290.78	117.81	407.56
	EXG	124.30	29.89	105.31	143.29	88.69	181.06
	CON	199.88	97.44	149.78	249.98	72.19	372.69
	total	194.16	94.01	165.92	222.41	72.19	407.56
VEGF II	EVG	225.28	83.77	180.64	269.92	116.69	398.94
	EXG	121.29	26.19	104.65	137.93	76.81	167.19
	total	180.71	83.21	148.45	212.98	76.81	398.94
VEGF III	EVG	237.16	80.30	194.37	279.94	143.69	389.31
	EXG	126.79	31.58	106.73	146.86	82.19	179.81
	CON	203.24	105.33	149.08	257.39	82.06	383.69
	total	194.91	91.78	167.34	222.49	82.06	389.31
VEGF IV	EVG	229.70	77.06	188.64	270.76	144.44	389.69
	EXG	129.14	35.56	106.54	151.73	81.94	201.31
	total	186.60	79.89	155.62	217.58	81.94	389.69

EVG—vibration training group; EXG—exercise without vibration group; CON—control group; vascular endothelial growth factor (VEGF); M—mean value; SD—standard deviation; 95% CI—upper and lower bound of the confidence interval; Min—minimum value; Max—maximum value; I–IV—measurement time points.

**Table 3:** Changes in VEGF, eNOS, hsCRP indices concentration after 12-week training programme in vibration training group (EVG) and exercise without vibration group (EXG).

Parameter		I	II	ΔII-I	III	IV	ΔIV-III	ΔIV-II	ANOVA *p* Values (η^2^)		
									Time	Group	G × T
VEGF [pg/mL]	EVG	240.48 ± 94.4 ##	225.28 ± 83.77 ##	−15.2 ± 38.24	237.16 ± 80.3 ##	229.7 ± 77.06 ##	−7.46 ± 11.87	4.41 ± 29.85	0.440 (0.104)	0.003 (0.003)	0.626 (0.069)
	EXG	124.3 ± 29.89	121.29 ± 26.19	−3.01 ± 20.33	126.79 ± 31.58	129.14 ± 35.56	2.34 ± 33.9	7.84 ± 31.3			
eNOS [U/mL]	EVG	43.46 ± 12.34	44.84 ± 12.12	1.39 ± 5.12	41.97 ± 11.58	42.49 ± 10.28	0.529 ± 7.191	−2.349 ± 3.943	0.145 (0.072)	0.315 (0.042)	0.909 (0.007)
	EXG	39.52 ± 7.2	41.15 ± 7.16	1.64 ± 9.1	37.68 ± 6.67	40.07 ± 7.57	2.39 ± 6.53	−1.09 ± 5.95			
hsCRP [mg/L]	EVG	0.93 ± 1.07	0.91 ± 1.11	−0.03 ± 0.35	0.89 ± 0.76	0.9 ± 0.78	0.01 ± 0.05	−0.01 ± 0.77	0.045 (0.072)	0.516 (0.016)	0.228 (0.056)
	EXG	1.05 ± 1.12	0.95 ± 0.89	−0.1 ± 0.41	1.3 ± 1.24	1.31 ± 1.27	0.01 ± 0,04	0.36 ± 0.49			

Values are mean * ±* standard deviation; η^2^ = eta squared; G × T: group-by-time interaction; vascular endothelial growth factor (VEGF); endothelial nitric oxide synthase (eNOS); high-sensitivity C-reactive protein (hsCRP); I—measurement performed before the first exercise training; II—measurement performed after the first exercise training; III—measurement performed before the last exercise training; IV—measurement performed after the last exercise training; ## Significant difference (*p* < 0.001) between the EVG/EXG.

**Table 4:** Changes in VEGF, eNOS, hsCRP indices concentration after 12-week training programme in vibration training group (EVG), exercise without vibration group (EXG) and at an interval of 3 months in control group (CON).

Parameter		I	III	Δ III-I	ANOVA *p* Values (η^2^)		
					Time	Group	G × T
VEGF [pg/mL]	EVG	240.48 ± 94.4 #	237.16 ± 80.3 #	−3.33 ± 38.84	0.874 (0.001)	0.003 (0.003)	0.843 (0.008)
	EXG	124.3 ± 29.89	126.79 ± 31.58	2.49 ± 28.08			
	CON	199.88 ± 97.44	203.24 ± 105.33	3.36 ± 35.2			
eNOS [U/mL]	EVG	43.46 ± 12.34	41.97 ± 11.58	−1.49 ± 7.47	0.275 (0.033)	0.613 (0.027)	0.973 (0.002)
	EXG	39.52 ± 7.2	37.68 ± 6.67	−1.84 ± 9.27			
	CON	43.93 ± 20.21	42.86 ± 17.46	−1.07 ± 8.05			
hsCRP [mg/L]	EVG	0.93 ± 1.07	0.89 ± 0.76	−0.05 ± 0.78	0.327 (0.023)	0.183 (0.078)	0.438 (0.039)
	EXG	1.05 ± 1.12	1.3 ± 1.24	0.25 ± 0.5			
	CON	0.56 ± 0.51	0.62 ± 0.57	0.06 ± 0.41			

Values are mean * ±* standard deviation; η^2^ = eta squared; G × T: group-by-time interaction; vascular endothelial growth factor (VEGF); endothelial nitric oxide synthase (eNOS); high-sensitivity C-reactive protein (hsCRP); I—measurement performed in rest on the first day of training programme; III—measurement performed in rest on the last day of training programme; # Significant difference (*p* < 0.05) between the EVG/EXG.

**Table 5:** Basic descriptive statistics of the eNOS variable.

		M	SD	95% CI	95% CI	Min	Max
eNOS I	EVG	43.46	12.34	36.33	50.58	27.12	65.59
	EXG	39.52	7.20	34.94	44.09	31.03	54.24
	CON	43.93	20.21	31.72	56.14	26.98	96.32
	total	42.40	14.14	37.82	46.98	26.98	96.32
eNOS II	EVG	44.84	12.12	37.85	51.84	28.17	61.09
	EXG	41.15	7.16	36.61	45.70	29.48	55.41
	total	43.14	10.12	39.05	47.23	28.17	61.09
eNOS III	EVG	41.97	11.58	35.28	48.65	25.03	59.64
	EXG	37.68	6.67	33.44	41.92	25.13	46.82
	CON	42.86	17.46	32.31	53.41	26.44	83.60
	total	40.94	12.65	36.84	45.04	25.03	83.60
eNOS IV	EVG	42.49	10.28	36.56	48.43	27.39	57.32
	EXG	40.07	7.57	35.26	44.88	30.72	55.20
	total	41.37	9.04	37.72	45.02	27.39	57.32

EVG—vibration training group; EXG—exercise without vibration group; CON—control group; endothelial nitric oxide synthase (eNOS); M—mean value; SD—standard deviation; 95% CI—upper and lower bound of the confidence interval; Min—minimum value; Max—maximum value; I–IV—measurement time points.

**Table 6:** Basic descriptive statistics of the CRP variable.

		M	SD	95% CI	95% CI	Min	Max
hsCRP I	EVG	0.93	1.07	0.36	1.50	0.20	3.90
	EXG	1.05	1.12	0.34	1.76	0.22	3.47
	CON	0.56	0.51	0.30	0.82	0.15	2.11
	total	0.82	0.92	0.55	1.10	0.15	3.90
hsCRP II	EVG	0.91	1.11	0.31	1.50	0.16	4.24
	EXG	0.95	0.89	0.38	1.52	0.26	3.24
	total	0.93	1.01	0.54	1.31	0.16	4.24
hsCRP III	EVG	0.89	0.76	0.49	1.29	0.18	2.49
	EXG	1.30	1.24	0.51	2.09	0.24	4.38
	CON	0.62	0.57	0.32	0.91	0.15	2.18
	total	0.90	0.88	0.63	1.16	0.15	4.38
hsCRP IV	EVG	0.90	0.78	0.48	1.32	0.18	2.49
	EXG	1.31	1.27	0.50	2.11	0.23	4.48
	total	1.07	1.02	0.68	1.47	0.18	4.48

EVG—vibration training group; EXG—exercise without vibration group; CON—control group; high-sensitivity C-reactive protein (hsCRP); M—mean value; SD—standard deviation; 95% CI—upper and lower bound of the confidence interval; Min—minimum value; Max—maximum value; I–IV—measurement time points.

## Data Availability

The data presented in this study are available on request from the corresponding authors.
